# Genomic Characteristics of Gender Dysphoria Patients and Identification of Rare Mutations in *RYR3* Gene

**DOI:** 10.1038/s41598-017-08655-x

**Published:** 2017-08-21

**Authors:** Fu Yang, Xiao-hai Zhu, Qing Zhang, Ning-xia Sun, Yi-xuan Ji, Jin-zhao Ma, Bang Xiao, Hai-xia Ding, Shu-han Sun, Wen Li

**Affiliations:** 1 Department of Medical Genetics, Second Military Medical University, Shanghai, 200433 China; 2 Department of Plastic Surgery, Changzheng Hospital, Second Military Medical University, Shanghai, 200433 China; 3 Center of Reproductive Medicine, Shanghai Changzheng Hospital, Second Military Medical University, Shanghai, 200003 China

## Abstract

Gender dysphoria (GD) is characterized by an incongruence between the gender assigned at birth and the gender with which one identifies. The biological mechanisms of GD are unclear. While common genetic variants are associated with GD, positive findings have not always been replicated. To explore the role of rare variants in GD susceptibility within the Han Chinese population, whole-genome sequencing of 9 Han female-to-male transsexuals (FtMs) and whole-exome sequencing of 4 Han male-to-female transsexuals (MtFs) were analyzed using a pathway burden analysis in which variants are first collapsed at the gene level and then by Gene Ontology terms. Novel nonsynonymous variants in ion transport genes were significantly enriched in FtMs (P- value, 2.41E-10; Fold enrichment, 2.8) and MtFs (P- value, 1.04E-04; Fold enrichment, 2.3). Gene burden analysis comparing 13 GD cases and 100 controls implicated *RYR3*, with three heterozygous damaging mutations in unrelated FtMs and zero in controls (*P* = 0.001). Importantly, protein structure modeling of the RYR3 mutations indicated that the R1518H mutation made a large structural change in the RYR3 protein. Overall, our results provide information about the genetic basis of GD.

## Introduction

Gender dysphoria (GD), a controversial diagnostic class in the American Psychiatric Association’s (APA) *Diagnostic Statistical Manual of Mental Disorders* (DSM) 5, causes persistent discomfort with one’s biologic sex. There are many other terms currently in use to describe this condition, including gender identity disorder, transsexual and transgender. It is estimated that fewer than 1 in 10,000 adult natal males and 1 in 30,000 adult natal females experience GD^1^. The etiology of GD is believed to be multifactorial, involving biological (including hormonal and genetic factors) and psychosocial factors^1, 2^. The brain is considered as the anatomical locus of gender identity^3, 4^. In human brains, there are region-specific dimorphisms with some structures larger in males (such as the hypothalamusand amygdala) and some larger in females (such as the caudate nucleus and hippocampus)^5^. However, it is still unclear what the role of these dimorphismsis in the development of GD. Heritability studies have demonstrated a genetic factor for the development of GD^6^. Recently, a twin study reported that 9 of 23 (39.1%) monozygotic female and male twins were concordant for GD. In contrast, none of the 21 same-sex dizygotic female and male twins were concordant for GD^7^. This indicates a role for genetic factors in the development of GD. A small number of candidate genes for GD have been studied, including the androgen receptor, estrogen receptor alpha, estrogen receptor beta, CYP17A1 and CYP19A1^8–13^. However, these results are rather mixed^4^. At present, no strong candidate gene has been identified that can account for the development of GD^1^.

It is generally considered, based on the small sample size, that replication of positive findings focusing on common polymorphic variants is difficult^14^. This raises the question of whether “some heritability” of rare complex diseases is missed^15^. For a substantial portion of these diseases, determining their heritability requires a more comprehensive assessment of rare human variants^16, 17^. The emergence of next-generation sequencing platforms, such as whole-genome sequencing (WGS) and whole-exome sequencing (WES), offers an unprecedented opportunity to identify the molecular causes of rare genetic diseases^18–20^. Many studies have identified genes conferring risk for spinal deformity^21, 22^, schizophrenia^23^ and autism^24^ by applying WES in patients with no family history of these disorders or their related phenotypes.

We hypothesized that rare genetic variants are a major contributor to GD susceptibility. In the current pilot study, we employed a combined multistep approach to examine 9 female-to-male transsexuals (FtMs) using WGS and 4 male-to-female transsexuals (MtFs) using WES to identify rare variants associated with GD. These genetic and clinical studies of GD patients were restricted to the Chinese Han population. The present study suggests the value of next-generation sequencing platforms in revealing genetic mechanisms involved in the development of GD, but also highlights their limitations and the need for additional studies using larger patient and control sample sizes.

## Results

**Figure 1:**
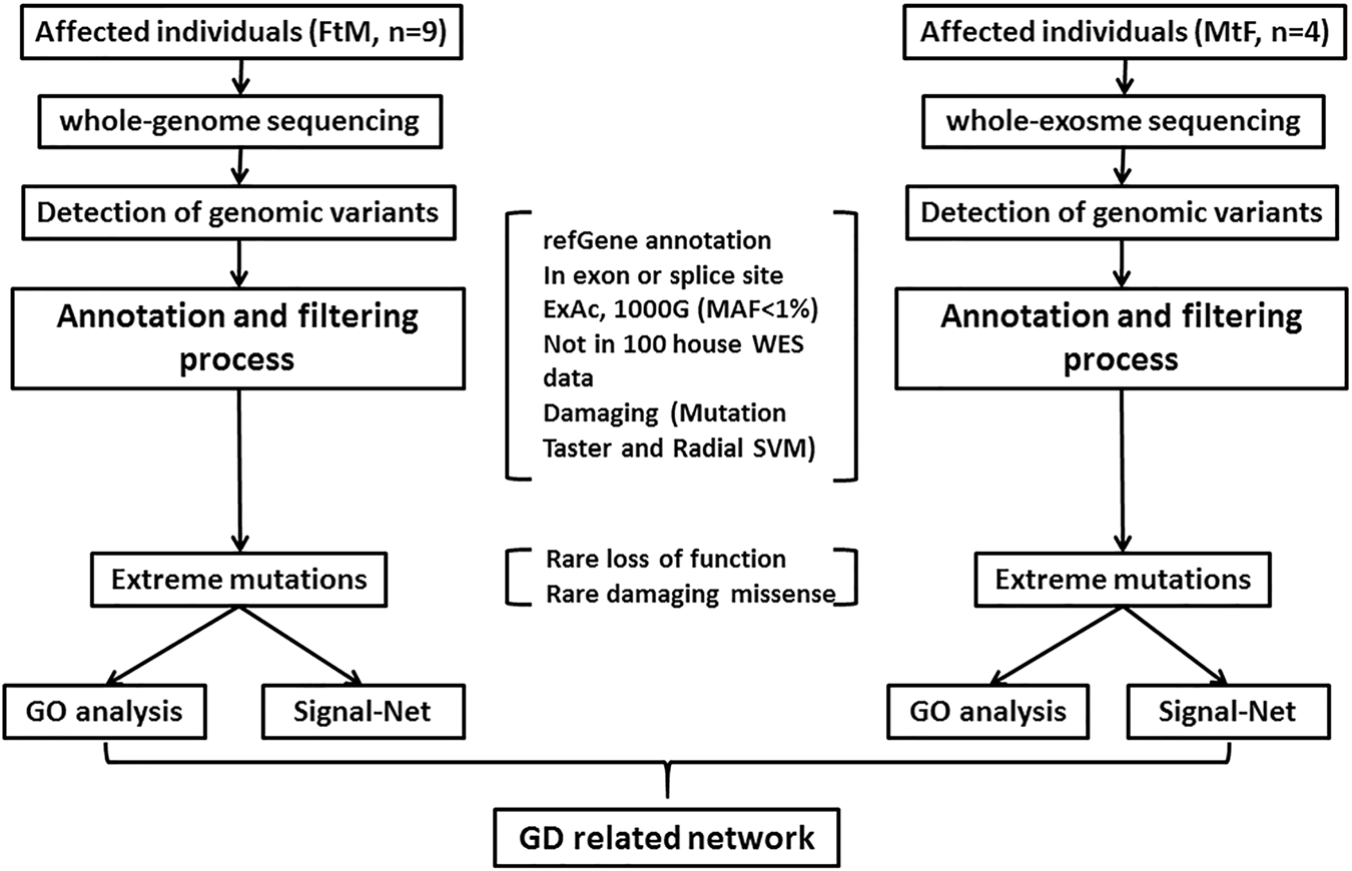
Flowchart for the exploration of disease genes in sporadic GD patients.

**Figure 2:**
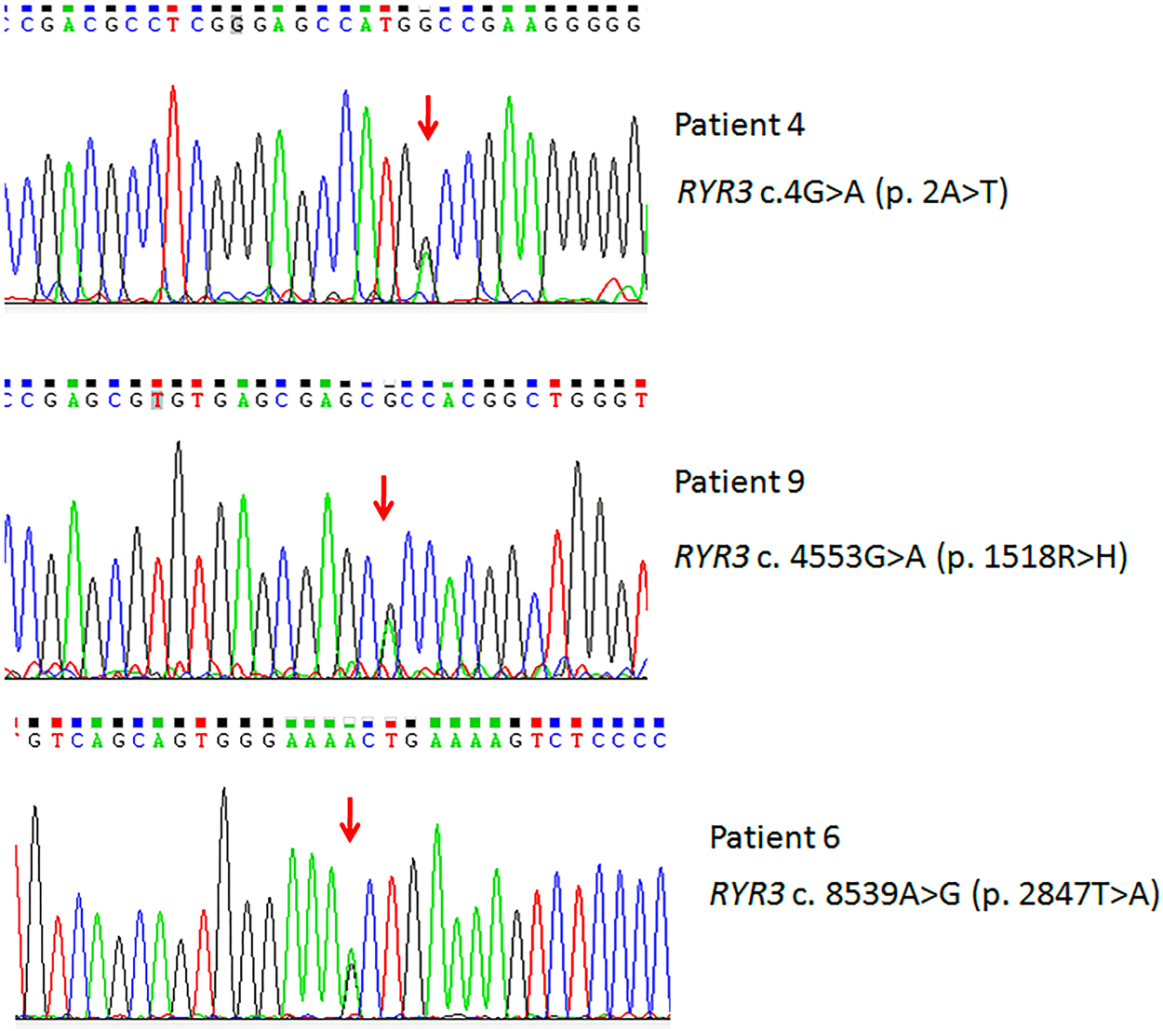
Heterozygotes *RYR3* mutations are identified in three FtMs (Patient 4, Patient 6, Patient 9). All mutations were verified by Sanger sequencing. Red arrows indicate the positions of point mutations.

We hypothesized that rare genetic variants are a major contributor to GD susceptibility. In the current pilot study, we employed a combined multistep approach to examine 9 FtMs using WGS and 4 MtFsusing WES toidentify rare variants associated with GD. Their mean age at sex reassignment surgery was 30.2 (±4.3) years. None of them have a family history of GD, schizophrenia or related phenotypes. All subjects (FtMs and MtFs) were Han Chinese and diagnosed as early-onset GD. Other clinical and demographic characteristics of the included subjects are shown in Table S1. Raw data for the whole genome screen are shown in Table S2. A total of 632,000,316–904,682,628 reads were generated. The average map rate was 96.68%, and approximately 77.31–90.20 Gbp were aligned to the reference genome (hg19) with a mapping quality of Q20. The information from the whole exome screen is shown in Table S3. On average, 97.7% of the generated sequences aligned to the reference genome (hg19), and 97.7% of the targeted bases in each individual were assessed by ≥5 independent sequence reads. Only rare variants (defined as minor allele frequency [MAF] < 1% in 1000 Genome, ExAC) causing nonsense, splice-site, missense or insertion/deletion mutations were included in the analysis. Furthermore, we excluded variants found in our own in-house exomes (n = 100) from individuals with unrelated diseases. All the filtered rare variants were predicted to be disease-causing by the mutation prediction software MutationTaster and RadialSVM. This filtering strategy was selected to enrich for mutations that are rare and most likely to be deleterious (Fig. 1). A total of 401 non-synonymous SNVs covering 377 genes (no indels) were identified in FtMs (Table S4), and 283 non-synonymous SNVs and 20 exonic indels covering 275 genes were identified in MtFs (Table S5). Among the mutated genes in GD patients, *Ryanodine Receptor 3* (*RYR3*), which is a ryanodine receptor that functions to release calcium from intracellular storage for use in many cellular processes, was found to be recurrently mutated in three FtMs and zero in 100 controls (gene burden analysis, *P* = 0.001, Table 1). In addition, all the three *RYR3* variants were confirmed using Sanger sequencing (Fig. 2), and none of 275 ethnicity-matched controls (130 male and 145 female Han Chinese) carried these variants.

**Table 1:** Summary of *RYR3* mutations in GD patients.

cDNA mutation*	Protein alteration	MutationTaster score	MutationTaster pred	RadialSVM score	RadialSVM pred	1000g_EAS	ExAC_EAS	Sample ID
c.4G > A	p. 2A > T	0.974	D	0.38	D	.	.	Patient 4 (FtM)
c. 4553G > A	p. 1518R > H	1	D	0.973	D	0.002	0.001	Patient 9 (FtM)
c. 8539A > G	p. 2847T > A	0.994	D	0.138	D	.	0.0031	Patient 6 (FtM)

*The accession number for *RYR3* is GenBank NM_001036.

We further analyzed the biological function of the 377 FtM mutated and 275 MtF mutated genes using the DAVID functional classification tool (DAVID 6.7 https://david-d.ncifcrf.gov/summary.jsp). For the 377 mutated genes in FtMs, the enriched GO terms were assigned to 67 clusters ranked by the biological significance of the gene groups based on GOTERM_BP_FAT (Table S6). Fifty-four genes included in the top cluster (enrichment score: 7.3) belong to 4 GO terms that are mainly involved in ion transport (Table S7). For the 275 mutated genes in MtFs, the enriched GO terms were assigned to 55 clusters ranked by the biological significance of the gene groups based on GOTERM_BP_FAT (Table S8). Twenty-six genes included in the top cluster (enrichment score: 2.1) belong to 5 GO terms that are mainly involved in ion transport (Table S7).

**Figure 3:**
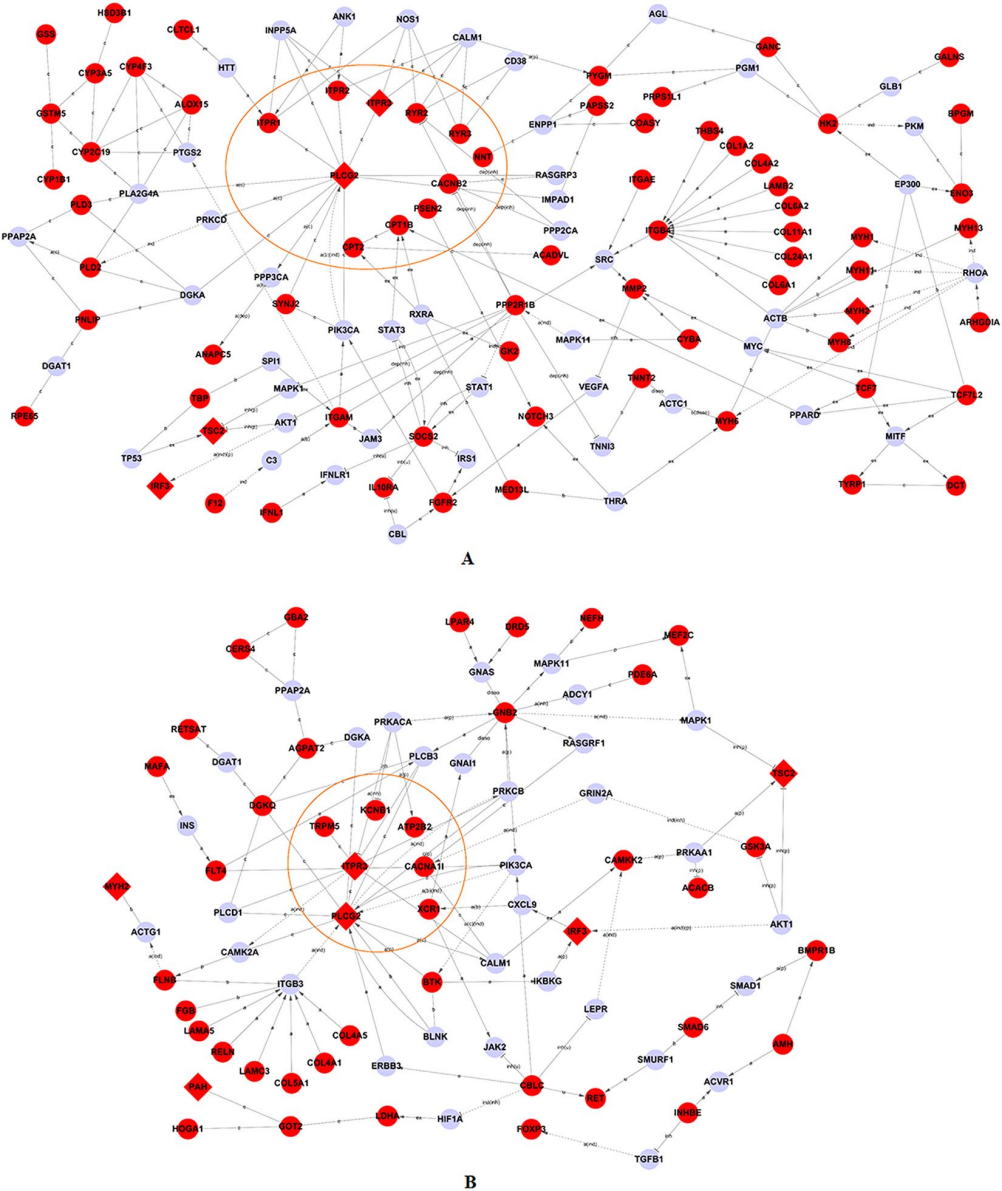
Functional analysis of the network containing identified rare and damaging mutations in GDs reveals enrichment for genes involved in ion transport. The network was algorithmically constructed using GeneSignalNetwork software (Genminix, Shanghai, China) based on the functional and biological connectivity of genes. The network is graphically represented as nodes (genes) and edges (the biological relationship between genes). Red nodes represent genes containing identified mutations in FtMs (**A**) and MtFs (**B**). The other nodes (light blue) are those that GeneSignalNetwork automatically includes because they are biologically linked to the studied genes based on evidence in KEGG. The genes in yellow circles participate in the process of ion transport.

Knowledge of molecular-level interactions between proteins has enabled the development of signal-net networks enriched for the 377 genes mutated in FtMs (Fig. 3A) or the 275 genes mutated in MtFs (Fig. 3B). The underlying connections were explored by MySQL 5.1.3 (Oracle, CA, USA) using experimentally verified interaction data from KEGG (http://www.kegg.jp/kegg/pathway.html). We found that the two networks (based on the mutated genes in FtMs and MtFs, respectively) encompass ion transport related modules. The ion transport-related module components in the FtM network (Fig. 3A) included 11 proteins: ITPR1, ITPR2, ITPR3, RYR2, RYR3, NNT, CACNB2, CTT1B, CPT2, PSEN2 and PLCG2. The ion transport-related module components in the MtF network (Fig. 3B) included 7 proteins: ITPR3, TRPM5, KCNB1, ATP2B2, CACNA1I, XCR1 and PLCG2.

**Figure 4:**
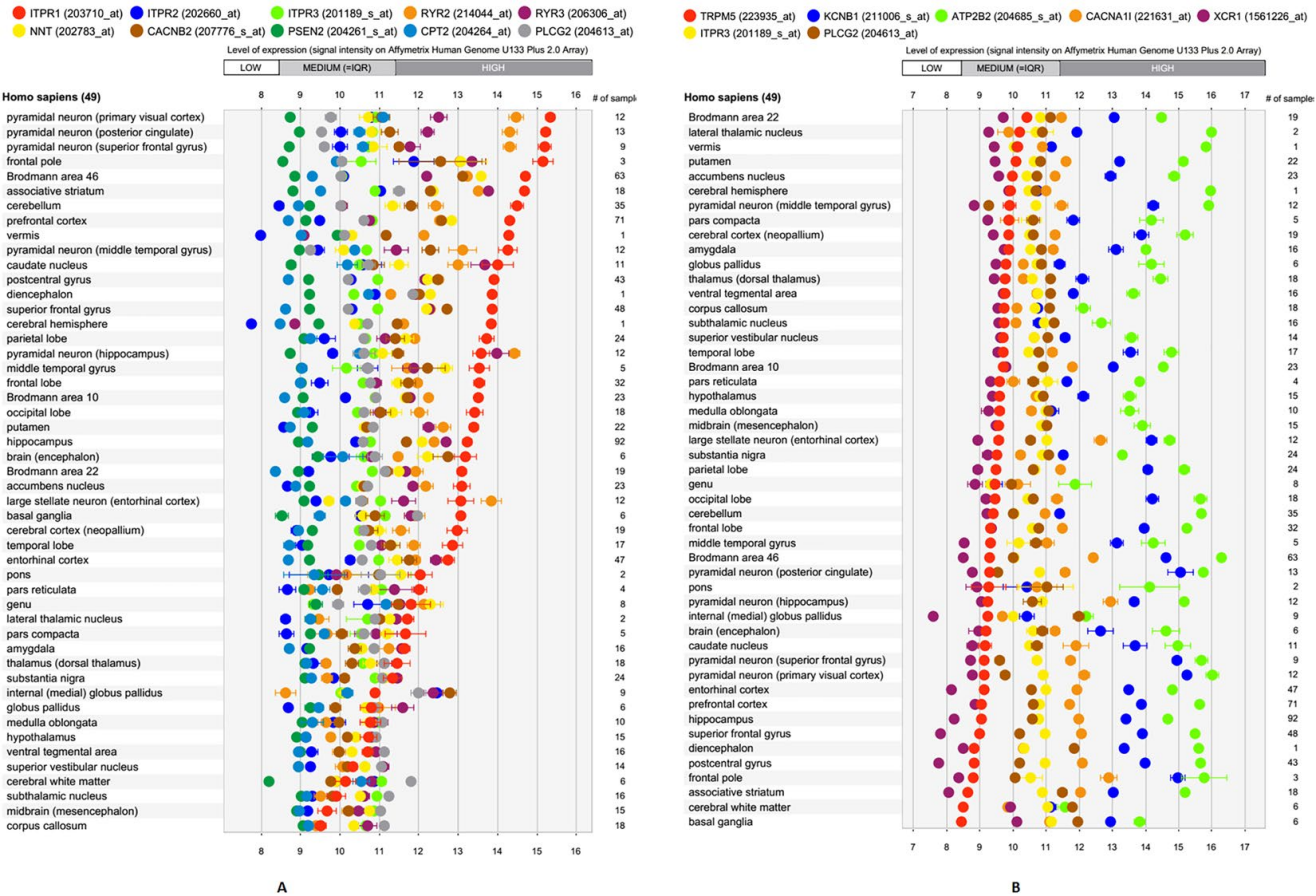
The relative expression levels of mutated ion transport genes in FtMs (**A**) and MtFs (**B**) in healthy brain tissue were identified using the GENEVESTIGATOR platform.

GD is commonly thought to arise from a discrepancy between cerebral and genital sexual differentiation. We therefore examined the expression of ion transport- related module components in different anatomical regions of the brainacross previously published microarray data sets using the GENEVESTIGATOR platform. The anatomy profile (Gene Atlas tool) results revealed strong RYR3 expression in the hippocampus, associative striatum, frontal pole and caudate nucleus (signal intensity on Affymetrix Human Genome U133 Plus 2.0 Array >13), but low or medium signals in other regions of the brain (signal intensity on Affymetrix Human Genome U133 Plus 2.0 Array <13) (Fig. 4A). However, PLCG2 and ITPR3 were uniformly expressedin each brain region (Fig. 4; for PLCG2, 9< signal intensity on Affymetrix Human Genome U133 Plus 2.0 Array < 12; for ITPR3, 10 < signal intensity on Affymetrix Human Genome U133 Plus 2.0 Array < 12).

**Figure 5:**
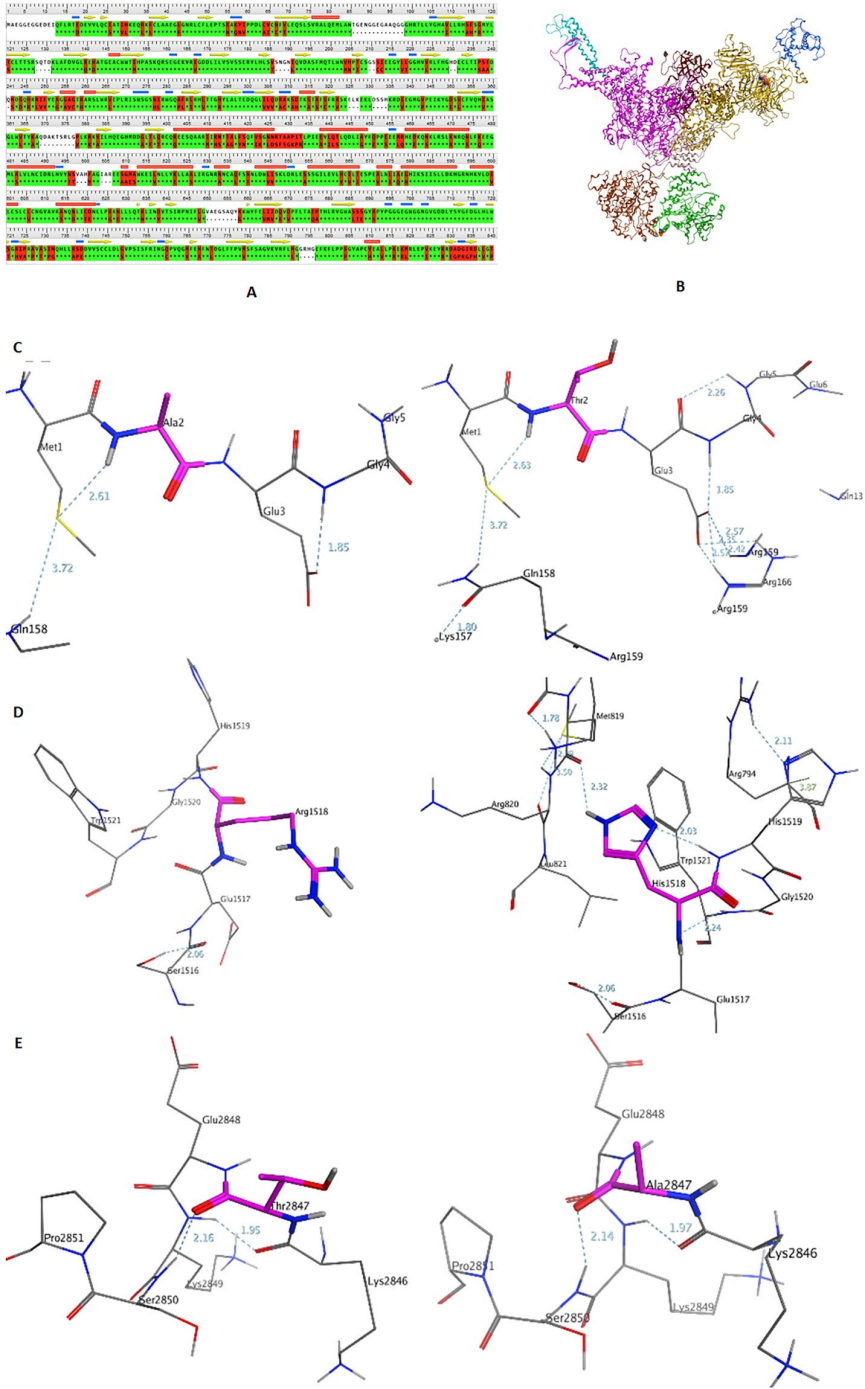
Analysis of conformational changes in mutant proteins. (**A**) The sequence alignment (part of the whole sequence) between *Homo sapiens RYR3* (up) and *Oryctolagus cuniculus RYR1* (down). The sequence identity is approximately 64.6%. (**B**) The homology model of the RYR3 receptor obtained by using MOE. The three mutation sites: A2, R1518 and T2847 are highlighted in the ball mode. Localized interactions at the three mutation sites: A2, R1518 and T2847. (left: **C,D**, and **E**) shows the original structures, T2, H1518 and A2847. (right: **C,D**, and **E**) shows the mutated structures. The three core residues are highlighted in purple and the hydrogen bonds formed around them are show.

Comparative modeling methods use structural templates that have the highest sequence homology with the target protein. Homologous proteins were identified by scanning the protein sequence of *Homo sapiens* RYR3, obtained from the ExPASy server, against 3D structures deposited in the Protein Data Bank using PSI-BLAST. The *Oryctolagus cuniculus* RYR1 (PDB code: 3J8H) was a promising template with sequence identity of 64.6% and a good *E*-value (Fig. 5A). The PDB structure was loaded into MOE and the sequence alignment performed in MOE with the alignment constrained between the target and the template.

A model, based on the template using a single template alignment approach, was generated using MOE software as described in the methods section. Ten intermediate models were generated and the final model was taken as the Cartesian average of all the intermediate models. Then, the final model was submitted to global minimization using the energy minimize module of MOE (Fig. 5B). To evaluate the quality of the modeled structure, a geometric analysis was performed in MOE, including a Phi-Psi Plot, bond lengths, bond angles, and rotamers. The stereochemical quality of the protein backbone and side chains was evaluated using Ramachandran plots via Phi-Psi Plot analysis. In the Ramachandran plot, 95% of the residues of the RYR1-based model were in the allowed region, which indicates that the backbone dihedral angles φ and ψ in the final model were reasonably accurate.

To obtain the stable structure, the homology model was minimized, followed by a manual mutation of p. 2A > T, p. 1518R > H and p. 2847T > A. MOE suggested possible changes in the molecular structure induced by these mutations. These mutations may introduce a new intramolecular hydrogen bond causing the structural changes. Of the three mutations, the p. 1518R > H mutation produced the largest structural change in the RYR3 protein. Figure 5D shows that the mutation caused three intramolecular hydrogen bonds to form between Met819, Gly1520, Trp1521 and His1518, which promots structural changing that move the side chain of the His1518 from the outside to the inner side. In the wild-type RYR3 homology model, Arg1518 did not show any interactions with these residues. This phenomenon was not seen in the p. 2A > T and p. 2847T > A mutants (Fig. 5C and E). In both the wild-type and p. 2A > T mutant, residue Ala2 and Thr2 could form a hydrogen bond with Met1 (Fig. 5C). The length of the side chains for Ala and Thr are almost the same. In both the wild-type and p. 2847T > A mutant, a hydrogen bond is found either between Thr2847 and Ser2850 or Ala2847 and Ser2850. In p. 2A > T and p. 2847T > A mutants, the structural changes may not be obvious.

## Discussion

It is widely accepted that genetic factors play key roles in the etiology of GD^1^. In recent years, the association between GD and gene polymorphismshas been investigated^8–13, 25, 26^. We investigated possible GD-associated pathogenic mutations by performing a WGS analysis of 9 FtMs and WES analysis of 4 MtFs. We identified heterozygous mutations of *RYR3* in three Han Chinese with GD. To the best of our knowledge, this is the first time that genotypic assessment by next generation sequencing has been applied to research GD. To use this new advanced technique of genetic analysis, it is best that the sample be homogeneous; therefore, all subjects (FtMs and MtFs) were Han Chinese and diagnosed as early-onset GD (Table S1).

Electrical activity in neurons requires a close functional coordination between plasmalemmal ion channels and ion transporters^27^. Although ion channels have been studied intensively for several decades, research on ion transporters is in its infancy. For brain disorders such as Parkinson’s disease, schizophrenia and depression the ion transporter system, being intertwined with many other signaling systems, plays a key role in pathogenesis and therapy^28–31^. Studying the functions of these transporters may lead to major paradigm shifts in our understanding of the mechanisms underlying brain development and plasticity in health and disease. GD is thought to be a consequence of atypical cerebral sexual differentiation^4^. The observed differences between GD patients and controls indicate that gender dysphoria may be associated with changes in multiple structures and involve a network (rather than a single nodal area)^32–35^. There is evidence indicating the important role of sex hormones in the sexual differentiation of brain^36, 37^. It is also believed that the foetal brain may develop into a male brain under the influence of testosterone, or into a female brain in the absence of this hormone^38^. Thus, GD may result from a discrepancy between sexual brain and genital differentiation caused by genetic or hormonal deviations^4^. Ryanodine receptors are intracellular calcium-release channels found on the endoplasmic reticulum of all cells. RYR3 is highly expressed in the hippocampus, associative striatum, frontal pole and caudate nucleus (Fig. 3A). During the physiological processes of sexual brain development, the intracellular calcium homeostasis may be largely regulated by RYR3. Mutations of *RYR3* may cause imbalance of intracellular calcium homeostasis, leading to impairment of neuronal function. Although our data show that specific ion transporter genes, including *RYR3*, may contribute to GD risk, it is equally striking to consider the combined impact of variants in ion transporter genes as a class on GD susceptibility. The majority of genes within this group may contribute to GD risk, but the risk may also increase proportionally with the number of variants within an individual. Demonstration of the collective effect of multiple rare variants across ion transporter genes on GD fits with a polygenic burden disease model that was previously described^22, 39^.

In summary, our results provide information about the genetic basis of GD. This study also has some limitations. First, because the subjects were of Han Chinese origin, the results cannot be generalized to all populations. Second, the study was performed in only 9 FtMs and 4 MtFs. Therefore, similar studies using larger numbers of patients are necessary.

## Materials and Methods

### Subjects

All the patients were diagnosed according to the Diagnostic and Statistical Manual of Mental Disorders, fifth edition (DSM-5) criteria for GD in adults. All blood samples were collected between March 2012 and March 2016 from GD patients before sex reassignment surgery. Clinical and demographic characteristics of the included subjects are shown in Table S1. The study was approved by the Second Military Medical University (Shanghai, China) ethics committeeand written informed consent was obtained from all participating patients. The methods were carried out in accordance with the principles stated in the Declaration of Helsinki.

### WGS and WES

Our analysis included WGS data from 9 FtMs and WES data from 4 MtFs. For WGS, DNA was extracted from blood, and 500 bp insert libraries were prepared according to the protocol provided by Illumina. The libraries were sequenced on HiSeq X platforms with paired-end reads of 101 bp. For sequence data analysis, read pairs were mapped with the Burrows-Wheeler Aligner (BWA), and the resulting files were converted to the pileup format via SAMtools^40^. After PCR duplications were removed, single nucleotide variations (SNVs) and indels were identified. Four MtFs were selected for WES. Whole-exome capture by the SureSelect Human All Exon Kit (Agilent) and high-throughput sequencing by the HiSeq. 2000 sequencer (Illumina Inc.) were conducted by Genergy (Shanghai, China).

### Bioinformatics Analysis

To explorethe potential biological functions of the mutated genes identified inFtMs and MtFs, the genes were uploaded to DAVID 6.7^41^ for functional annotation clustering analysis based on GO terms (GOTERM_BP_FAT).

The GeneSignalNetwork analysis was performed by Genminix (Shanghai, China) as previous described^42^. First, an interactions repository derived from KEGG was constructed. Using GeneSignalNetwork, we obtained a given protein’s upstream or downstream interactions through the whole KEGG Pathway database. We connected two gene nodes when their corresponding proteins were either directly or indirectly connected by a linker gene in the interaction network. The network for each gene was measured by counting the number of upstream and downstream genes or binding genes, which were expressed as in-degree and out-degree or degree, respectively (More information can be found in this link. http://college.gcbi.com.cn/fangfaxue/676.jhtml).

For in silico identification of the expression levels of genes in different anatomical regions of the brain, we used the Signature tool from the GENEVESTIGATOR searchengine^43^. For analysis, we selected data sets from the Affymetrix Human Genome U133 Plus2.0 Array platform. Then, knowledge-drivenselection of healthy brain microarray data sets wasperformed.

### Homology Modeling

The target sequence of wild-type *Homo sapiens* Ryanodine receptor 3 (RYR3) was selected as the input for homology modeling. The crystal structure of the Ryanodine receptor 1 from *Oryctolagus cuniculus* (PDB code: 3J8H, resolution: 3.8 Å) was downloaded from the Protein Data Bank for use as the template. The homology modeling process was performed using the Molecular Operating Environment package, version 2015.1001 (Chemical Computing Group, http://www.chemcomp.com). Ten independent intermediate models were built by permutational selection of various loop candidates and side-chain rotamers. The intermediate model that scored best according to the GB/VI scoring function was chosen as the final model.

### In Silico Mutation Modeling

To analyze the possible changes in the molecular structure, in silico mutations were performed based on the wild-type structure obtained from the homology modeling. The mutations in RYR3 (A2T, R1518H, andT2847A) were modeled using the Protein Builder application in MOE. The stable structures of the wild-type and mutant RYR3 proteins were obtained by minimization in MOE. Amber10:EHT was used as the force field. The intramolecular interactions and structural changes were analyzed both in the wild-type and mutant RYR3 proteins.

## Electronic supplementary material

Supplementary Information

Table S4

Table S5

Table S6

Table S8
