# Supplementary material for: Genomic Characteristics of Gender Dysphoria Patients and Identification of Rare Mutations in RYR3 Gene
Source: Sci Rep. 2017 Aug 21;7:8339. doi: 10.1038/s41598-017-08655-x (PMC5567086; doi:10.1038/s41598-017-08655-x)
Supplement: Supplementary file 1 — Supplementary Information [file 41598_2017_8655_MOESM1_ESM.doc]

**Genomic Characteristics of Gender Dysphoria Patients and Identification of Rare Mutations in *RYR3* Gene**

Fu Yang^1,*, #^, Xiao-hai Zhu^2,*^, Qing Zhang^3^, Ning-xia Sun^3^, Yi-xuan Ji^3^, Jin-zhao Ma^1^, Bang Xiao^1^, Hai-xia Ding^3^, Shu-han Sun^1,#^ , Wen Li^3,#^

^1^Department of Medical Genetics, Second Military Medical University, Shanghai, 200433, China; ^2^Department of Plastic Surgery, Changzheng Hospital, ^3^Center of Reproductive Medicine, Shanghai Changzheng Hospital, Second Military Medical University, Shanghai, 200003, China;

^*^These authors contributed equally to the manuscript.

^#^Address reprint requests to: Shu-Han Sun, Ph.D., Department of Medical Genetics, Second Military Medical University, Xiangyin Road 800, Shanghai200433, China. E-mail: shsun@vip.sina.com; fax:+00-86-21-81871053; Wen Li, M.D., Center of Reproductive Medicine, Shanghai Changzheng Hospital, Second Military Medical University, Fengyang Road 415, Shanghai 200003, China. E-mail:lyyliwen@sina.com; fax:+00-86-21-81886711; or Fu Yang Ph.D., Department of Medical Genetics, Second Military Medical University, Xiangyin Road 800, Shanghai200433, China. E-mail: yangfusq1997@smmu.edu.cn; <tel:+00-86-21-81871058>;

**Table S1. Characteristics of patients**

| **Sample ID** | **Age**  **(years)** | **Assigned gender** | **Psychiatric diagnosis** | **First**  **symptom** | **Psychological counseling**  **effect** | **Karyotype analysis** | **Sexual orientation** | **Primary and secondary sex characteristics** | **Family history** | **Sex hormone detection** |
| --- | --- | --- | --- | --- | --- | --- | --- | --- | --- | --- |
| Patient 1 | 29 | Female | Early-onset GD | Strong preference for cross-dressing | Yes  in vain | 46XX | homosexual | Normal | No | Normal |
| Patient 2 | 29 | Female | Early-onset GD | Strong preference for cross-dressing | Yes  in vain | 46XX | homosexual | Normal | No | Normal |
| Patient 3 | 30 | Female | Early-onset GD | Strong preference for cross-dressing | Yes  in vain | 46XX | homosexual | Normal | No | Normal |
| Patient 4 | 29 | Female | Early-onset GD | Strong preference for cross-dressing | Yes  in vain | 46XX | homosexual | Normal | No | Normal |
| Patient 5 | 29 | Female | Early-onset GD | Strong preference for cross-dressing | Yes  in vain | 46XX | homosexual | Normal | No | Normal |
| Patient 6 | 44 | Female | Early-onset GD | Strong preference for cross-dressing | Yes  in vain | 46XX | homosexual | Normal | No | Normal |
| Patient 7 | 27 | Female | Early-onset GD | Strong preference for cross-dressing | Yes  in vain | 46XX | homosexual | Normal | No | Normal |
| Patient 8 | 29 | Female | Early-onset GD | Strong preference for cross-dressing | Yes  in vain | 46XX | homosexual | Normal | No | Normal |
| Patient 9 | 30 | Female | Early-onset GD | Strong preference for cross-dressing | Yes  in vain | 46XX | homosexual | Normal | No | Normal |
| Patient 10 | 25 | Male | Early-onset GD | Strong preference for cross-dressing | Yes  in vain | 46XY | homosexual | Normal | No | Normal |
| Patient 11 | 29 | Male | Early-onset GD | Strong preference for cross-dressing | Yes  in vain | 46XY | homosexual | Normal | No | Normal |
| Patient 12 | 32 | Male | Early-onset GD | Strong preference for cross-dressing | Yes  in vain | 46XY | homosexual | Normal | No | Normal |
| Patient 13 | 30 | Male | Early-onset GD | Strong preference for cross-dressing | Yes  in vain | 46XY | homosexual | Normal | No | Normal |

**Table S2. Overview of genome sequencing data for all Ft**Ms passing quality control

| Sample | Total reads | MapRate | Duplication removed reads | Total mapped bases (/Gb) | Average depth (X) | Coverage | | | | | |
| --- | --- | --- | --- | --- | --- | --- | --- | --- | --- | --- | --- |
|  |  |  |  |  |  | ≥1X | ≥5X | ≥10X | ≥20X | ≥30X | ≥40X |
| Patient 1 | 874,148,308 | 96.05% | 764,157,484 | 80.00 | 27.96 | 98.66% | 98.23% | 97.73% | 88.14% | 39.97% | 6.54% |
| Patient 2 | 853,540,380 | 95.68% | 757,826,141 | 79.80 | 27.89 | 98.68% | 98.24% | 97.72% | 88.79% | 39.81% | 5.77% |
| Patient 3 | 904,682,628 | 96.27% | 802,527,592 | 85.08 | 29.74 | 98.67% | 98.24% | 97.80% | 92.73% | 50.80% | 8.70% |
| Patient 4 | 719,289,752 | 96.84% | 651,798,854 | 87.11 | 30.44 | 98.75% | 98.44% | 98.08% | 94.08% | 52.70% | 7.10% |
| Patient 5 | 632,000,316 | 97.16% | 577,111,170 | 77.31 | 27.02 | 98.74% | 98.41% | 97.94% | 87.92% | 31.00% | 2.51% |
| Patient 6 | 725,443,860 | 97.02% | 657,616,110 | 87.83 | 30.69 | 98.76% | 98.45% | 98.10% | 94.13% | 54.38% | 7.96% |
| Patient7 | 690,030,108 | 97.11% | 625,875,096 | 83.77 | 29.27 | 98.76% | 98.44% | 97.99% | 90.36% | 46.00% | 6.71% |
| Patient 8 | 700,983,956 | 96.91% | 636,798,668 | 84.91 | 29.67 | 98.73% | 98.42% | 98.05% | 93.20% | 47.89% | 5.64% |
| Patient 9 | 709,556,036 | 97.08% | 631,998,758 | 90.20 | 31.52 | 98.75% | 98.46% | 98.15% | 95.29% | 59.68% | 9.47% |

**Table S3. Overview of exome sequencing data for all MtFs passing quality control**

| *Variable* | Patent 10 | Patent 11 | Patent 12 | Patent 13 |
| --- | --- | --- | --- | --- |
| Total reads (millions) | 84.1 | 83.7 | 68.0 | 94.4 |
| % of aligned reads | 97.9% | 97.6% | 97.7% | 97.6% |
| % of on-target reads | 76.4% | 76.0% | 75.6% | 76.0% |
| % of on Target +- 150 Regions Covered by Reads | 97.8% | 97.4% | 97.5% | 97.9% |
| Mean coverage | 107.4X | 106.4X | 85.8X | 119.8X |
| % of target bases covered with 0~5X | 1.7% | 1.8% | 2.3% | 1.5% |
| % of target bases covered with 5~10X | 1.8% | 1.9% | 2.5% | 1.6% |
| % of target bases covered with 10~20X | 4.1% | 4.4% | 6.4% | 3.6% |
| % of target bases covered with 20~30X | 5.6% | 5.9% | 8.8% | 4.6% |
| % of target bases covered with 30~40X | 7.1% | 7.3% | 10.1% | 5.9% |
| % of target bases covered with 40~50X | 8.1% | 8.2% | 10.3% | 6.9% |
| % of target bases covered with >50X | 71.1% | 70.0% | 59.2% | 75.4% |

**Table S7**. DAVID functional enrichment analysis results (Annotation Cluster 1)

| **Go terms** | **Count** | **Fold enrichment** | **P-value** | **Genes** |
| --- | --- | --- | --- | --- |
| **FtM mutated genes** | | | | |
| ion transport | 47 | 2.8 | 2.41E-10 | SLC22A16, CLCN3, SLC5A4, CPT2, KCNC3, SLC22A13, SLC22A15, SLC9A2, CACNB2, VDR, SCN9A, SLC30A2, KCNG4, OCA2, SCN10A, GRID1, ANO10, CPT1B, SLC12A8, SLC12A1, SLCO4C1, NOX5, TRPC6, SLCO4A1, SLC12A4, SCN2A, SLC24A5, SLC34A1, ITPR3, ATP13A5, ITPR1, ITPR2, ATP13A4, TRPM1, CNGA2, SLC26A4, NNT, SLC26A7, RYR3, CLIC5, KCNN2, PSEN2, CLIC6, RYR2, KCNH8, KCNH4, ATP7B |
| transmembrane transport | 38 | 3.1 | 1.88E-09 | SLC22A16, CLCN3, SLC5A4, CPT2, KCNC3, SLC22A13, SLC22A15, SLC37A3, SLC9A2, SCN9A, ABCB10, SLC30A2, KCNG4, OCA2, SCN10A, CPT1B, SLC12A8, SLC12A1, ABCB8, TRPC6, SLC12A4, SCN2A, SLC24A5, ABCC12, ITPR3, ABCB6, ITPR1, ITPR2, TRPM1, CNGA2, SLC25A30, SLC26A4, SLC26A7, RYR3, RYR2, KCNH8, KCNH4, ATP7B |
| cation transport | 33 | 2.8 | 3.19E-07 | SLC22A16, SLC5A4, CPT2, KCNC3, SLC22A13, SLC9A2, CACNB2, VDR, SCN9A, SLC30A2, KCNG4, SCN10A, CPT1B, SLC12A8, SLC12A1, TRPC6, SLC12A4, SCN2A, SLC24A5, SLC34A1, ITPR3, ATP13A5, ITPR1, ITPR2, ATP13A4, NNT, RYR3, KCNN2, PSEN2, RYR2, KCNH8, KCNH4, ATP7B |
| metal ion transport | 26 | 2.6 | 2.62E-05 | KCNC3, SLC5A4, SLC9A2, CACNB2, VDR, SCN9A, SLC30A2, KCNG4, SCN10A, SLC12A8, SLC12A1, TRPC6, SLC12A4, SCN2A, SLC24A5, SLC34A1, ITPR3, ITPR1, ITPR2, RYR3, PSEN2, KCNN2, RYR2, KCNH8, KCNH4, ATP7B |
| **MtF mutated genes** | | | | |
| ion transport | 26 | 2.3 | 1.04E-04 | TCIRG1, TRPM5, CLCN2, KCND1, KCNB1, CACNA1I, HEPHL1, ITPR3, TCN1, SLC34A3, HTR3E, ATP2B2, SLC26A6, BEST1, KCTD18, SLC5A9, SLC4A7, SLC4A9, SLC5A7, XCR1, HTR3A, PDZK1, ARL6IP5, PKDREJ, KCNH5, ATP5L2 |
| cation transport | 17 | 2.1 | 0.006 | TCIRG1, KCND1, KCNB1, CACNA1I, HEPHL1, ITPR3, TCN1, SLC34A3, ATP2B2, KCTD18, SLC5A9, SLC4A7, SLC5A7, XCR1, PDZK1, KCNH5, ATP5L2 |
| metal ion transport | 14 | 2.0 | 0.017 | ATP2B2, KCND1, KCNB1, CACNA1I, KCTD18, SLC4A7, SLC5A9, HEPHL1, SLC5A7, ITPR3, XCR1, TCN1, SLC34A3, KCNH5 |
| monovalent inorganic cation transport | 10 | 2.2 | 0.040 | TCIRG1, KCND1, KCNB1, SLC4A7, KCTD18, SLC5A9, SLC5A7, SLC34A3, KCNH5, ATP5L2 |
| transmembrane transport | 8 | 1.7 | 0.066 | TCIRG1, CLCN2, TRPM5, KCND1, KCNB1, CACNA1I, ITPR3, TCN1, SLC26A6, SLC5A9, SLC5A7, PDZK1, KCNH5, ATP5L2 |
